# Mechanisms of Antifungal Properties of Metal Nanoparticles

**DOI:** 10.3390/nano12244470

**Published:** 2022-12-16

**Authors:** Yael N. Slavin, Horacio Bach

**Affiliations:** Department of Medicine, Division of Infectious Diseases, University of British Columbia, 410-2660 Oak St., Vancouver, BC V6H3Z6, Canada

**Keywords:** nanoparticles, metals, ROS, mechanism of defense, fungi, transcriptomics, proteomics, gene regulation, antifungal resistance

## Abstract

**Highlights:**

**What are the main findings?**

- Mechanisms by which nanoparticles exert their damage on fungi were described.
- Transcriptomic studies regarding exposure to nanoparticles were analyzed.

**What is the implication of the main finding?**

- Potential applications and future venues were discussed.
- More studies are needed to determine the safety of future applications of metallic nanoparticles.

**Abstract:**

The appearance of resistant species of fungi to the existent antimycotics is challenging for the scientific community. One emergent technology is the application of nanotechnology to develop novel antifungal agents. Metal nanoparticles (NPs) have shown promising results as an alternative to classical antimycotics. This review summarizes and discusses the antifungal mechanisms of metal NPs, including combinations with other antimycotics, covering the period from 2005 to 2022. These mechanisms include but are not limited to the generation of toxic oxygen species and their cellular target, the effect of the cell wall damage and the hyphae and spores, and the mechanisms of defense implied by the fungal cell. Lastly, a description of the impact of NPs on the transcriptomic and proteomic profiles is discussed.

## 1. Background

Fungi were initially included as a part of Kingdom Plantae but in 1969 were grouped into Kingdom Fungi, which comprises diverse groups with different morphologies, such as unicellular yeasts and multicellular organisms [1]. However, since then, there have been many changes in classification within and even at the Kingdom level. For example, in 1981, oomycetes were moved to a novel Kingdom termed Kingdom Chromista due to new phylogenetic studies [2].

The exact values of fungal diversity have yet to be discovered, with numbers of species ranging from 2.2–3.8 million [3], up to 5.1 million [4], and 6 million [5], among others. While the species diversity ranges in the millions, around 8000 species are plant pathogens, and only a fraction infects humans. Nonetheless, fungal diseases are responsible for more than 1.6 million deaths yearly, a number over 3-fold higher than malaria estimates and comparable to tuberculosis [6]. Furthermore, the high number of fungal plant pathogens introduces more issues such as (1) loss of biodiversity (e.g., chytridiomycosis as a dominant cause of amphibian extinction) [7], and (2) blights that destroy crops (e.g., rice blast fungus, causing up to 35% rice harvest loss across 85 countries) [8].

The rate of antifungal resistance development [9] has been called “unprecedented”. This is because immunocompromised individuals are at a higher risk of fungal infections than healthy individuals. Moreover, medical advancements over the past few decades (e.g., antibiotic discovery, cancer treatment progress, surgical transplants) and the HIV epidemic have increased the number of immunocompromised people, which has, in turn, shifted fungal infections from being an infrequent cause of disease to being an important contributor to human morbidity and mortality worldwide [10]. There are six antifungal drug classes (see below), and this scarcity, combined with the increasing resistance, has led to the need for novel treatments.

One promising solution as an antifungal agent is the use of nanoparticles (NPs). Many studies investigate this field of nanotechnology, but the mechanisms of action involved in NPs use as an antifungal remain widely unknown. This review summarizes and discusses proposed mechanisms of action, including reactive oxygen species (ROS) production, cell wall architecture, plasma membrane deformation, interaction with fungal structures, inhibition of spore germination, and gene and protein regulation. This review also discusses the formulation of NPs using primary literature focused on NP synthesis for antifungal testing.

## 2. Fungi Groups

Generally, all fungi may be split into two groups: yeasts or molds (filamentous fungi). Dimorphic fungi, such as *Candida albicans*, can grow in either form depending on environmental conditions, such as undergoing morphogenesis to engage in pathogenic behavior. Yeasts are unicellular organisms that typically reproduce asexually through binary fission or budding but uncommonly reproduce sexually, usually in high-stress environments such as that void of nutrients. Molds are multicellular organisms comprised of hyphae filaments that branch out as the fungus grows, responsible for enzyme release and nutrient absorption. Usually, hyphae that are composed of cells separated by walls called septum (septa in plural) are termed “septate hyphae” but can also lack walls and cell membranes between the compartments, termed “coenocytic hyphae” or “non-septate hyphae”. Collectively, a network of hyphae is called mycelium. If a mycelium grows in an aggregated form, this aggregation is called a pellet. During the budding process of yeast, when daughter cells stick to the parent cell, and a small structure is formed, this is termed “pseudohyphae” [11]. Molds reproduce sexually or asexually through the formation of spores such as conidia and sporangiospores, which are types of asexual spores. As the primary goal of spores is to be dispersed, they experience a period of dormancy and have different compounds coating them for adaptation to stressful environments [12,13].

## 3. Fungal Cell Wall Structure

The cell wall is critical for fungal growth, defense, morphogenesis, and biofilm formation. It functions to buffer fluctuations of osmotic pressure, sense external stimuli, and protect against detrimental conditions such as dryness, heat, toxic molecules, etc., while protecting from microbes. The cell wall is also crucial for pathogenicity and virulence in pathogenic fungi as it will aid in invasion while protecting the fungus from host defense mechanisms [14]. However, the distinctive structure makes it a good target for antifungal agents.

**Figure 1:**
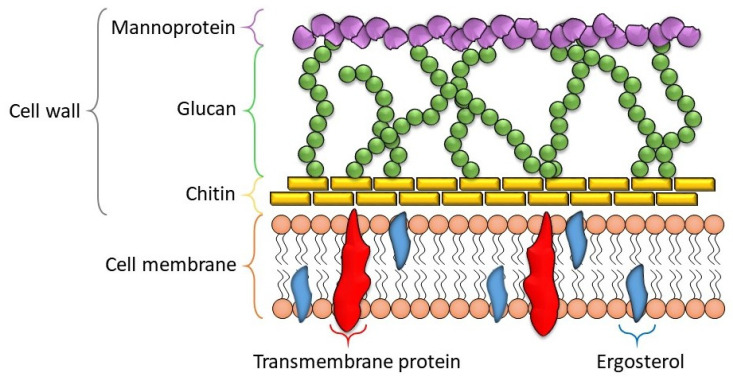
Fungal cell wall organization and membrane.

The cell wall (Figure 1) contains polysaccharides and proteins such as chitin, glucan, mannan, glucanases, and glycoproteins. The cell wall may appear to be a rigid structure, but it is dynamic, constantly being remodeled due to proliferation (expansion, sporulation, and branching) and environmental challenges [15]. Events such as binary fission or expansion of hyphae require the simultaneous activities of anabolic and catabolic enzymes to remodel the structure as a whole completely.

A brief description of the main components of the fungal cell wall is provided below.

## 4. Polysaccharides

### 4.1. Glucan

**Figure 2:**
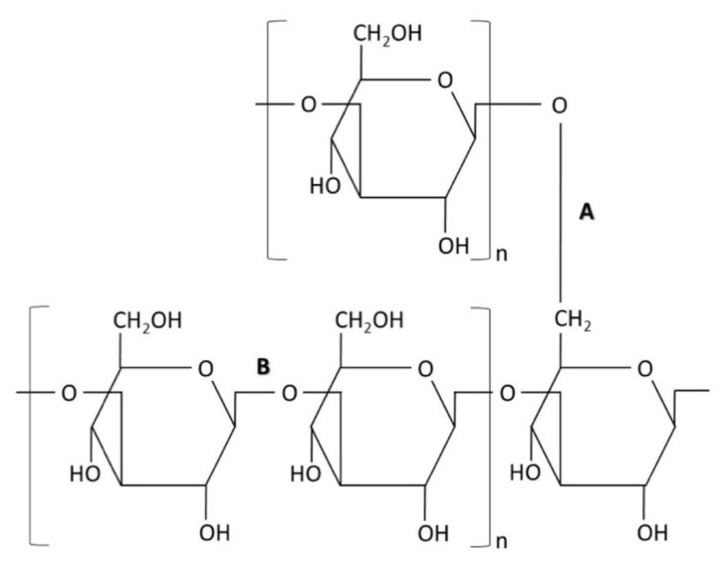
Glucan structure shows the (**A**) β-1,6 linkages and (**B**) β-1,3 linkages.

This polysaccharide (Figure 2) is the major component of the fungal cell wall, and it is synthesized by the polymerization of glucose monomers (approximately 1500 residues) through covalent β-1,3-linkages [16,17,18]. Alternate linkages occur at the C6 position of the linear glucan, where branching of the polysaccharide occurs through β-1,6 linkages of approximately 50 glucose monomers [19,20,21]. The glucan synthesis is mediated by membrane-bound glucan synthases that transfer a glucosyl residue to the chain through a β-1,3 bond in a vectorial synthesis [19,22,23]. Additionally, the branched glucans provide a point of cross-linking to other cell wall components, such as chitin and proteins [24].

### 4.2. Chitin

**Figure 3:**
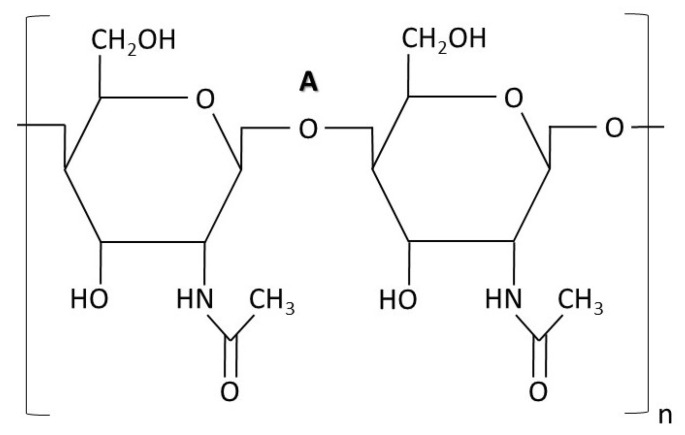
Chitin structure of polymerized N-acetylglucosamine monomers with (**A**) β-1,4 linkages.

This polysaccharide (Figure 3) is synthesized by polymerizing N-acetylglucosamine monomers, which are linked as β-1,4-linkages. The chitin synthesis is linear, and the formation of microfibrils has been observed. Moreover, these microfibrils might condensate and crystallize extracellularly. It represents a total of 1–2% and up to 20% of the total dry weight of yeast and filamentous fungi, respectively, [25]. Chitin provides tensile strength to the cell wall; when disrupted, fungal cells undergo morphological changes accompanied by an osmotic sensitivity [26].

## 5. Proteins

The proteins on the fungal cell wall are mainly glycoproteins which are extensively modified with N-linked and O-linked oligosaccharides. Their linkage to different oligosaccharide chains depends on the fungal species. For instance, yeast such as *Saccharomyces cerevisiae* and *C. albicans* synthesize mannoproteins, which are the product of glycosylation of the proteins with mannose polymers or mannans [15]. Other filamentous fungi, such as *Neurospora crassa* and *Aspergillus fumigatus,* contain a hybrid of galactose and glucose, forming galactomannan structures [27,28,29].

A family of five proteins, called GAS, is anchored onto the membrane by a phosphoglyceride glycosylphosphatidylinositol (GPI) [30]. Interestingly, the deletion of the *GAS1* gene caused morphological defects in *S. cerevisiae* [31] and is likely associated with its transglycosylase function [32].

The cell wall undergoes a dynamic rearrangement due to the activity of multiple enzymes. These enzymes are orchestrated during the remodeling of the cell wall, including chitinases and glucanases. This battery of proteins hydrolyzes and rearranges the skeleton of the cell wall before the enzymatic activity of glycosyltransferases, which will finish the synthesis and cross-linking of the polymers [33,34].

### 5.1. Glucanases

As a component of the cell wall, glucans change as a result of the constant remodeling of the cell wall. For instance, *S. cerevisiae* contains an array of approximately 15 endo- and exo-1,3-β-glucanases with crucial functions during proliferation and transglycosylation activity [35]. Interestingly, the deletion of endoglucanases caused cell clumping, supporting that these enzymes are involved in the digestion of septa [35]. Similar enzymes have been found in other yeasts, such as *C. albicans* and *Schizosaccharomyces pombe,* and similar phenotypes have been observed upon their deletion compared to *S. cerevisiae* [36].

Endoglucanases have also been reported in filamentous fungi, exhibiting similar glucanase and transglycosidase activities. For example, a dual function was attributed to glucanases in *A. fumigatus*, which cleave 1,3-β-linkages in glucan with a concomitant transfer to a non-reducing end of another 1,3-β-glucan molecule, leading to an elongation of the chain catalyzed by the enzyme glucan elongating glucanosyltransferase or GEL1. This enzyme is also anchored to the membrane by GPI molecules and showed homology to GAS1 in the yeasts [32].

### 5.2. Chitinases

Chitin synthases are responsible for the production of chitin. The genome of *S. cerevisiae* encodes three chitin synthases termed CHS1p, CHS2p, and CHS3p [37]. CHS1p is involved in cell repair during the proliferation of the fungi, CHS2p is involved in synthesizing the septa [38,39], whereas CHS3p generates the rest of the chitin found in the cell [40,41]. Chitin is crucial for cell survival, and deleting the three chitin synthases is lethal for *S. cerevisiae* [39,42].

Other groups of fungi possess a higher number of chitin synthases. For instance, *A. fumigatus* has seven genes responsible for chitin polymerization, and their deletions from the genome are not necessarily lethal for growth, but they exhibit morphological changes [43,44]. In the case of *N. crassa*, four chitin synthases are present in the genome but are also predicted to have three additional isoenzymes [45,46,47,48,49]. As in *A. fumigatus*, individual deletions of these chitin synthase genes showed different phenotypes, such as slowing growth accompanied by a change in morphology [45].

Under specific physiological events, endochitinases remove chitin, allowing cell wall remodeling. For example, during the duplication of yeast, the newly formed cells (mother and daughter) are separated from each other by hydrolysis, which is mediated by an endochitinase [50,51,52]. These chitinases are also GPI-anchored to the cell membrane. Deleting the *CHIA* gene encoding a chitinase in *A. nidulans* affected the spores and decreased the growth rate of the hyphae [53].

## 6. Nanoparticle Formulation as Antifungal Agents

There is a need for novel antifungal treatments as currently available options are lacking. It is worth noting that the idea of increased potential future resistance is worrying as fungi are eukaryotes, as are their most common hosts found in Kingdoms Animalia and Plantae. That is to say, the eukaryotic hosts of pathogenic fungi have similarities in metabolism and protein structure, and finding targets to differentiate organisms becomes more complicated [54].

**Figure 4:**
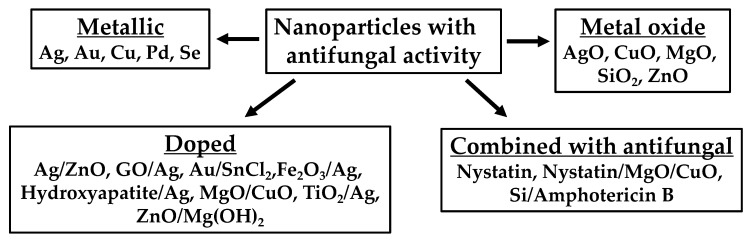
Different types of metallic NPs developed against fungi.

Nanotechnology has rapidly progressed over the past few decades and using NPs as potential antifungals have been an expanding field of interest. The present study summarized the literature from 2005 until 2022 regarding the NP formulations that showed antifungal activity (Table 1, Table 2, Table 3, Table 4, Table 5 and Table 6). A summary of the different types of metallic NPs studied is shown in Figure 4.

NPs have unique physical and chemical properties compared to their larger counterparts because their small size leads to a high surface-to-volume ratio. NPs can be produced using the top-down technique, where small particles are formed from a more significant part, or the bottom-up technique, where small particles assemble from smaller pieces. The bottom-up approach is typically favored for the synthesis of NPs as it makes more homogeneous products with fewer defects and has parameters that are easier to control during fabrication. For example, altering compound surface charges can facilitate bottom-up processes such as interfacial electrostatic self-assembly [63]. As another example, the electrochemical synthesis of metal NPs can be utilized to avoid reducing chemicals and control size dispersion based on the cell electrode potential tuning [80]. This is interesting because usually, a bottom-up process requires a metal salt and a reducing agent, such as sodium borohydride or Sn(II) chloride [77,92].

There is no standardized approach for the fabrication of NPs, and they can be found in various shapes, sizes, compositions, and formulations. However, spherical AgNPs are the most popular. Ag has been known for centuries to possess antimicrobial properties [93]. We know today that AgNPs possess these same properties and unique optical, electrical, and other physical/chemical properties. It is proposed that a potential mechanism of toxicity of Ag comes from its ion release coupled with its catalytic oxidation abilities [94].

Combining different AgNPs can exhibit a synergistic effect [95]. For example, combinations of AgNPs with other oxides, such as maghemite (γ-Fe_2_O_3_) or magnetite (Fe_3_O_4_), can lead to NPs that possess the unique Ag features while incorporating the magnetic features included in the iron oxide compounds, such as controllable alterations through magnetic field manipulation [67]. Moreover, combining AgNPs with graphene-oxide (GO) produced nanosheets with a three- and a seven-fold increase in bacterial inhibition efficiency over their counterparts in one study [63]. However, in another, combining AgNPs with ineffective copper NPs (CuNPs), a synergistic effect was not measured but somewhat diminished the activity of the AgNPs [96].

Other types of oxides have been investigated as well. A study looking at the antifungal activity of metal(loid) oxides found that Al_2_O_3_, Mn_3_O_4_, SiO_2_, and SnO_2_ reduced cell viability in a dose-dependent manner, whereas In_2_O_3_ showed no toxicity, even at concentrations of 100 mg/L [97]. GO is also of interest and has been used in functionalization and investigated as it is a single-atom-thick and a 2D sp^2^-bonded carbon lattice with a large surface area [98]. This is important because a lattice will innately prevent aggregation as dispersion will be enhanced and remain during the usage of the particles. Thus, GO’s surfactant-like properties allow it to attach metal NPs to hyphal interfaces, forming nanosheets with concomitant mechanical damage due to their extremely sharp edges [63].

Other oxides, such as titanium dioxide (TiO_2_) NPs, have shown that the mechanism of antimicrobial ability requires visible light to affect [99,100]. The photocatalytic activity is based on hydroxyl radical generation. It is known that photocatalysis works through photons exciting electrons to the conduction band and forming electron-hole pairs. An alternative to increase the radical hydroxyl production is doping TiO_2_ with Ag, potentially by accepting the photoinduced electrons and holes with an increase in the degradation efficiency [100]. Another study found that undoped TiO_2_NPs did have some antifungal properties, but the combination with Ag enhanced their efficacy. Fungal species also play a role in the antifungal activity of NPs, e.g., *Venturia inaequalis* was more affected by undoped TiO_2_ than *Fusarium solani*. Still, the most effective nanomaterial was Ag-doped hollow TiO_2_NPs. However, this may have been because the hollow formulation was spherical, and the solid formulation shape was indiscernible in TEM imaging [100]. As shown in another study, TiO_2_NPs did not show activity unless doped with nitrogen (N) and fluorine (F) [99]. Lastly, zinc oxide (ZnO) NPs are a prevalent oxide formulation [101,102,103,104,105].

Green synthesis is a method of synthesizing NPs that is more biologically- and environmentally friendly. This is done by using biogenic sources such as plants, bacteria, or fungi and incorporating their naturally occurring innately-antimicrobial metabolites or compounds into or onto NPs, acting as stabilizers or removing the need for harsh chemical components [106,107]. Sometimes, the color of the NPs may change when using plant extracts to synthesize NPs. For instance, a change to red/brown may indicate the incorporation of saponins and phenolic compounds as stabilizing agents providing the reduction power during production [108]. In another study, encapsulated *Cymbopogon martinii* (ginger grass) essential oil (extracted through hydro distillation) rather than using plant extracts during formulation in chitosan NPs to exploit the oil’s antifungal properties against *Fusarium graminearum* [106].

NPs made with biogenic sources [61,62,71,83,105,107,108,109,110] can have an enhanced effect compared to NPs made only chemically [105]. The biogenic formulation can increase the antifungal effect via increased reactive oxygen species (ROS) production, cell membrane disintegration, spore reduction, gene expression changes, and mycelial destruction [61,71,72,103,105]. Upon altering parameters, biogenic sources may produce AgNPs less effective than their chemical-only counterparts. An important parameter is a biological source; for example, the use of the fungus *Penicillium chrysogenum* as part of the synthesis of AgNPs was more effective than *A. oryzae*, although still less effective than the chemical-only process [62]. The formulation process may differ from organism to organism; however, the metal ions are typically entrapped by or surrounded by compounds released from the microorganism and undergo reduction.

Since fungal culture is a lab-controlled environment, the parameters for the formulation of NPs are also controlled [111]. For example, He et al. hypothesized that during gold NPs (AuNPs) formation, the bacterium *Rhodopseudomonas capsulate* secrets NADH- and NADH-dependent enzymes. During the electron transfer from NADH by NADH-dependent reductase, Au (III) ions capture electrons and, as a result, are reduced to Au (0) [112]. This mechanism of NADH- and NADH-dependent enzymes is likely also present in fungi, as extracellular filtrate of *F. oxysporum* strains used for synthesis contained NADH-dependent nitrate reductase enzymes [113].

Fungal biomolecules can behave as stabilizing agents during the formulation process and help achieve spherical particles [62,107]. For instance, during AuNPs formulation, it was found that biomolecules >3 kDa were not able to reduce Au (III) to Au (0). Interestingly, when comparing the AuNPs formation ability of various fungal extractions, it was found that while extractions containing large biomolecules were not capable of forming NPs, others containing small components would make unstable NPs, likely due to the small components’ inability to act as stabilizing agents. Combining different fractions developed stable NPs with slightly increased sizes of ~30 nm, compared to previous sizes of ~8–30 nm. Thus, it was concluded that biomolecules <3 kDa, such as glucose or amino acids, are involved in reducing the metal, and biomolecules >3 kDa, such as proteins, are involved in the stabilization [107].

## 7. Effect of the Size and Shape on the NPs Activity

Alongside the formulation process, the resulting shape and size are crucial to the activity. The shape is difficult to compare as most formulated particles are spherical, and amongst other forms, there are a variety of sizes and compositions. One study comparing CuNPs vs. Cu nanowires found that minimum inhibitory concentration (MIC) depended on the fungal strain. Still, mechanisms of action were different between the two samples [66] and are discussed in further depth later in this review. One study directly compared NPs shaped as spheres, cubes, and wires. Cubes were the most effective antifungal, followed by spheres and wires. Ag formulations were more effective than Au formulations, and the material seemed to matter more between spheres and cubes, but no wires. For example, Ag spheres were more effective than Au cubes in most cases. However, Ag wires were not. This may also be due to sizing, as the wires were 250–500 nm, while all other NPs were 30–50 nm. However, the shape was crucial for recovery as resistant isolates of *Candida*, when exposed to the nanocomposites, began to develop after 48 h of exposure to wires or spheres, but not with cubes, regardless of composition [76].

Size is often one of the most important factors to consider. Typically smaller NPs are found to have more potent antifungal activity than larger NPs [66,69,77,80,86,114,115,116], potentially due to the higher surface-area-to-volume ratio, which can increase binding at various target sites, facilitates diffusion, and have a smaller tendency to aggregate [77,86]. NPs can decorate fungal spores whereas microparticles cannot, and when considering this decoration ability, the size factor is more important than surface functionalization, material, or charge [117]. In addition, combining NPs can result in an antagonistic effect, which is hypothesized to be due to increased particle size, potentially through aggregation [69]. Nonetheless, some experiments find NPs of different sizes to have comparable activity. However, it is worth noting that although outside the typical NPs range, NPs as large as 729 nm have exhibited antifungal activity [85,108].

Capping agents can be used in NPs formulation for increased consistency and stability while preventing undesired changes such as aggregation. This is not to be confused with reducing agents, which are necessary for synthesis through electron donation, not stability. Two types of reagents containing O and N atoms are used. These atoms should be able to interact with the Cu (II) ion and then prevent aggregation through steric hindrance.

Capping agents function by creating an interface between the NPs and the medium in which the NPs are synthesized [118]. Capping agents can also be used to prevent oxidation, such as the positive detergent cetrimonium or cetyl bromide (CTAB) in the formulation of CuNPs to prevent oxidizing damage from the air [80]. CTAB is a quaternary ammonium salt containing a polar group (NCH_3_)_4_^+^ and a C16 hydrocarbon chain, which enhances surface activity. The prevention of Cu oxidation follows the two-phase adsorption of the CTAB on the surface of the CuNPs. In the first phase, the bromide anions adsorb on the surface of the positively charged CuNPs, with a posterior interaction between the (NCH_3_)_4_^+^ cation to the bromide anion. When CTAB molecules adsorb on the CuNPs, the long chain *n*-cetyl can cover the more available surface [118].

Polymeric coatings are of interest as they can control the release of metal species due to a stabilizing shell. Because NPs loading and release are linked with antimicrobial activity, these coatings can manipulate antimicrobial efficacy [92]. For example, GO capped with polyvinylpyrrolidone (PVP) was functionalized with polydiallyldimethylammonium chloride (polyDDA), rendering GO positive at 33 mV. This positive charge facilitated assembly with negatively charged AgNPs, forming a GO-AgNPs nanocomposite [92].

PVP is commonly used in the synthesis of AgNPs [62,65,115,118]. The lone pairs from the oxygen and nitrogen atoms are donated to the *sp* orbitals of the Ag ions, which form a complex that ultimately facilitates reduction and nucleation due to the increased negative charge [119]. Colloidal Ag solutions without stabilizing agents lead to low Ag content, and the addition of PVP usually increases Ag concentration, except at higher concentrations (6 g/L), where particle aggregation is induced through deterring diffusion and decreasing electrostatic repulsion [120].

The combinations of the capping agent PVP mixed with stabilizers such as sodium lauryl sulfate (Na-LS or SDS), sodium naphthalene sulfonate (Na-NS), or sodium dodecyl benzenesulfonate (SDBS) were tested in NPs [120]. Absolute zeta potential was >30 mV in SDS NPs and SDBS NPs, indicative of stability in the colloidal suspension [121]. At the same time, Na-NS NPs had a zeta potential of −18.2 mV and varying size results indicative of sedimentation. SDS NPs were most effective at fungal growth inhibition when tested against a mold colony [122]. On the other hand, AgNPs with SDS showed a significantly increased MIC and minimum fungicidal concentration (MFC) against yeasts when compared to no stabilizers, or AgNPs stabilized with the surfactants Tween-80, Brij, or PVP 360, although all surfactants increased the activity of AgNPs likely due to their ability to disrupt cell walls by themselves [59]. It is worth noting that SDS has antifungal properties alone as well due to bonds to the plasma membrane through lipid and protein interactions that lyse the cell [123]. This accounts for the synergistic effect with NPs as this would allow the penetration of NPs, increasing fungal sensitivity. SDS has also been shown to inhibit the ATPase activity by interfering with P-glycoprotein function [124], an antifungal mechanism used by select antifungal drugs such as itraconazole, hydroxyitraconazole, posaconazole, isavuconazole, anidulafungin, caspofungin, and micafungin [125].

## 8. Antifungal Classes and Combination with NPs

Currently, there are six antifungal drug categories, including four main antifungal drug classes: allylamines, azoles, echinocandins, and polyenes. Some literature will include the antimetabolite class [125,126] and, more recently, triterpenoids, such as ibrexafungerp, the first drug in the triterpenoid class [125,127].

Ergosterol and β-(1,3)-D-glucan are favored targets in antifungals, as these molecules are crucial for the survival of pathogenic fungi. These compounds are attractive because they are not produced by human cells (Table 7) [125]. Ergosterol is essentially the cholesterol equivalent in fungi and protozoa. Found in the cellular membrane, it is responsible for the membrane’s integrity and flexibility. Ergosterol is structurally similar to cholesterol aside from containing a double bond and additional methyl group in the alkyl side chain; this trans double bond means it is not saturated like cholesterol. There is also a second double bond at 7,8-position, alongside the 5,6-positioned double bond found in the cholesterol [128].

As mentioned earlier, the second compound, β-(1,3)-D-glucan, is a fungal cell wall component.

Antifungal resistance is a problem that is increasing worldwide. This can arise due to many mechanisms, including gene upregulation [129] for cell wall component synthesis [130] or efflux pump synthesis [131], modification of target site [132], or development of biofilm [133], amongst others. For example, the overexpression of *ERG11* in yeast or *CYP51* in mold confers resistance through the overproduction of lanosterol 14-α demethylase, aiding cell wall building and maintenance [130,134]. Because NPs have numerous mechanisms of action, fungi would have to evolve in multiple ways to acquire resistance while maintaining homeostasis and survival. As it is difficult to combat the simultaneous antifungal mechanisms of NPs, even though some are similar to antifungals (e.g., gene regulation), it is unlikely that fungi would be able to become resistant, at the very least, not at the same rate as the current antifungals used today.

NPs can be synthesized and combined with antifungals to increase one or both of their antimycotic capabilities [61,73,81,109,110,135]. One study decorated SiNPs with amphotericin B (AmB) to create particles with antifungal ability that could adhere to surfaces and be reused up to 5 times. The NPs alone was 3–33 times less effective than AmB on their own, but the addition of the antifungal gave the NPs an antifungal ability stronger than that of 10 nm colloidal Ag [73]. This, paired with the ability to coat surfaces, makes the particles worth further investigation, particularly in coating medical devices. While many studies have shown the synergism of NPs combined with antifungal medications, the effect can vary based on which fungal species are being treated [109,110].

The addition of antifungals to NPs can also benefit efficacy in unexpected ways. It can increase the roughness of NPs surfaces, which may account for mechanical damage or increased surface areas [81]. When antifungals are enclosed in NPs rather than coated, this can reduce their toxicity, such as in the case of AmB, where hemolytic activity in mammalian red blood cells was reduced from approximately 66% to 30% [136]. Therefore, combining NPs with antifungals can enhance activity, alter the morphology of NPs, and reduce cytotoxicity in human cells. Also, NPs on their own can have stronger antimycotic abilities than traditional antifungals [65].

## 9. Mechanisms of Fungal Cell Damage

### 9.1. Membrane Damage

Exposure to NPs causes changes in the fungal cell wall, including surface shrinkage, cell aggregation, pit and pore formation, and general deformation [55,60,65,70,95,104,137]. Through microscopy, it has been detected that NPs may have direct contact and embedment within fungal cell walls during adsorption, which induces morphological change. Inner membranes also suffer distortion, with altered organelle disposition (such as increased intracellular vesicle and vacuole count) and decreased cytoplasmic content [65,86,114]. Severe damage causing pits or cell wall rupture is not guaranteed upon exposure to AgNPs. Still, even without this damage, AgNPs can be found surrounding and adsorbing onto cell walls. Ag dots in the cytoplasm can be detected using energy-dispersive X-ray spectroscopy (EDS). This could be smaller NPs, but also Ag ion penetration that ends in the formation of NPs intracellularly through the organic compound reduction [138]. Difficulty differentiating the cytoplasm/cell wall/plasma membrane borders after exposure to NPs is expected as the NPs disrupt these structures and blur boundaries [70,86,138]. The loss of intracellular structure fills the cell with cytoplasm and a seeming lack of organelles.

Observations of cell wall components show changes upon exposure to NPs. For instance, chitosan NPs encapsulating *C. martinii* essential oil and ZnO NPs caused a dose-dependent decrease in ergosterol and chitin, respectively [104,106]. AgNPs caused alterations of phosphatidylcholine-to-phosphatidylethanolamine ratios in treated cells, causing a loss of membrane integrity and cell function. Additionally, fewer C18:2 phospholipids were present in treated cells and inhibited desaturation ability [61]. Other nanocomposites, such as MgO/CuO sodium alginate microspheres with antifungal nystatin, showed that exposure to *C. albicans* damaged the cell membrane and leaked intracellular components. After 24 h, 94% of cells were in the late apoptotic stage, and proteins and DNA had seeped through the broken cell wall [81]. AgNPs also produce this effect, where fungal cell-free filtrate contains DNA after exposure [105].

The cell leakage in *C. albicans* cells leads to increased intra- and extracellular levels of glucose and trehalose, which can have protective functions during stress conditions and function as a carbon source [139]. Similar effects were found compared to AmB [55]. Through Raman spectroscopy, ZnO NPs were also found to increase the levels of carbohydrates and nucleic acids in the pathogenic fungus *Botrytis cinerea*, potentially indicating the need for the protective mechanism that carbohydrates (e.g., trehalose) can provide for the cell [102,139]. Moreover, when fungal growth was inhibited entirely, carbohydrate and nucleic acid levels decreased [102]. Interestingly, the exposure to ZnO NPs did not change protein and lipid levels in fungi, indicating that perhaps the cell wall was not impacted, and the particles may have been internalized, causing intracellular effects more severe than the cell wall/extracellular effects, or the cell wall was affected. Still, mechanisms for cell wall synthesis were altered to compensate for any loss.

Increased glucose levels were also seen in *C. albicans* and *S. cerevisiae* after the exposure to AgNPs; however, the decrease in glucose uptake was likely associated with a G1 cell cycle delay or ROS production. When treated with N-acetylcysteine (NAC), a ROS scavenger that increases antioxidant-reduced glutathione levels (GSH), glucose uptake was restored in *C. albicans* only. Therefore ROS production might play a role in reducing glucose uptake by *C. albicans* [115]. Combining the treatment of AgNPs with a glycolysis inhibitor such as bromopyruvate exhibits a synergistic effect on cell death in *C. albicans* but not in *S. cerevisiae* [115]. These effects could also be because *C. albicans* is pathogenic, whereas *S. cerevisiae* is not. Then if any pathways for pathogenicity and virulence are being targeted, only one strain will be affected.

ROS partake in lipid peroxidation, which can induce cell wall damage. N, F co-doped TiO_2_ NPs capture light and generate ROS, mainly hydroxyl radicals. These radicals attack the monomers of the cell wall, cleaving the glycosidic linkage and creating pores, leading to fungal death. Ethidium bromide (EtBr) was used to confirm cell wall damage, as EtBr can penetrate the cell and intercalate with DNA to give off signals under excitation only if the cell wall has been damaged [99].

Similarly, CuNPs induced significant cell wall damage in yeast cells. The cytoplasm membrane was detached from the cell wall, and organelles appeared swollen. Along with this cellular collapse, fimbriae (hair-like protrusions on the cell wall) decreased in number. Other Cu nanocomposites, such as Cu nanowires, also decreased fimbriae quantity. While yeast cells experienced enlargement and some cytoplasm leakage with cell wall disruption, most cells conserved their shape and cell wall integrity [66]. This indicates that although the MIC of nanowires and NPs were comparable, the mode of antimycotic activity was not the same. One of the reasons can be attributed to the presence of sharp edges in the NPs, suggesting that the cell wall damage occurred due to mechanical damage. Thus, although cell wall damage is common amongst NPs, this happens in different ways, including ROS production, gene regulation resulting in changes in cell wall components, mechanical damage, internalization, and removal of protective compounds.

### 9.2. DNA Interactions

Smaller NPs may cause fluid-phase endocytosis, bypassing the need for any cell wall damage [65]. It was shown that AgNPs could damage the cell wall and enter the cell, but at a smaller size can also enter without much damage. The antimycotic injury occurs at an intracellular level, including mitochondrial fragmentation, ribosome depolymerization, and chromatin damage. This triggers the production of multivesicular bodies [60]. Indeed, cell wall damage can cause DNA leakage out of the cell, but once NPs are inside the cell, some can intercalate with nucleic acids intracellularly.

AgNPs caused DNA condensation and fragmentation in *C. albicans* nuclei. These damages were validated by adding thiourea and its protective abilities against oxidative stress [140]. This indicates that although Ag ions can bind to DNA (negatively charged), which may play a factor in DNA destruction, oxidative stress also has a role. Additionally, damage can occur through the contact of NPs with DNA. AuNPs caused DNA damage directly by nuclear condensation and DNA fragmentation in *C. albicans*, which may account for cellular dysfunction and apoptosis [140]. After ZnO treatment, the increased nucleic acid production in *B. cinerea* may indicate the mechanism of NPs known in bacteria, where NPs can interact with the gyrase-DNA complex. This would encourage the fungal cell to produce more nucleic acid as compensation [102].

On the other hand, CuO NPs induced dose-dependent DNA-strand breaks with an effect increasing after an additional week of incubation. Two factors enhanced this effect: (1) the exposure of Cu^2+^ rather than NPs, and (2) the effect increased in fungi isolated from streams without metal pollution vs. streams with metal pollution [141]. The efficacy of the Cu ion form may be due to size, and effectiveness based on fungal strain is likely due to metal adaptation.

In summary, the fragmentation of DNA, which is enclosed in the nucleus, implies that the NPs should cross the nuclear membrane or produce significant damage in the intracellular membranes that would allow them to be in contact with the DNA. Moreover, it might also be the results of the ions released by the NPs that can affect the nuclear membrane at a distance.

### 9.3. Ion Release

Ion release has increased antimicrobial activity with an extended-release over time, improving results [63]. Some studies have shown that ions are more toxic than their NPs counterparts [141,142]. This could be due to size, which may facilitate penetration into cells, or the ability to complex with other biomolecules, such as proteins, nucleic acids, negatively-charged lipids, etc. In arsenic-resistant *Aspergillus* ssp., thiol compounds were found to play a significant role in detoxification. Because Ag ions are complexed with thiols, ion release may interfere with a fungal detoxification process [143], as thiols in the cytoplasm are essential to keep reducing conditions in this compartment. Thus, ROS produced by the effect of ions released by the NPs exposed to an oxidative environment would exhaust the levels of thiols, altering the intracellular redox potential. As a result, cells might activate the apoptosis program [144]. Another study suggested that producing Cu^2+^ paired with cell wall destruction through mechanical means allows ions to behave as a biocide, interacting with cellular compounds [66]. However, a study found that using NaCl neutralized the efficacy of Ag, indicating that ions are partially responsible for its antifungal ability [145]. This neutralization can result from a change in the electrolyte balance with the activation of different ion pumps. But this phenomenon might not happen in applying NaCl as an accompanying therapy because it might affect the isotonic balance and cause indirect damage to other eukaryotic cells.

To account for ion activity, supernatants of NPs were tested, and it was found that SiO_2_ NPs toxicity was due to NPs alone, Al_2_O_3_ NPs and SnO_2_ NPs toxicity was due to a combination of NPs and ions, and Mn_3_O_4_ NPs toxicity was primarily due to ion activity [97]. Ultimately, fewer studies about ion release look at fungi rather than bacteria. Still, from the ones available, it is evident that ion release is sometimes an NP mechanism of action for antimycotic activity.

### 9.4. Damage to Hyphae and Spores

NPs can have severe impacts on fungal hyphae and spores. Fungi treated with GO-AgNPs, AgNPs, MgO NPs, ZnO NPs, or CuNPs showed hyphae deformation, appearing distorted and shrunken [63,69,95,96,105,114]. NPs change the growth patterns, clumping and thinning hyphal fibers [60,70,86].

Interestingly, even when CuNPs did not impact fungal growth, hyphae still appeared damaged [96]. As a result of hyphal damage, NPs can inhibit mycelial growth, often dose-dependent manner [65,86,95,112,146]. Sub-inhibitory AgNP treatment in *C. albicans* inhibited the growth of mycelia after morphogenesis was triggered. Observations of the mycelia showed that it did not extend nor form around the presence of AgNPs, but untreated control had a healthy mycelial formation [147]. SiO_2_ NPs could reduce mycelial radial growth up to 100% at 100 µg/mL exposure. They behaved dose-dependently, impacting mycelium fresh and dry weight [63]. ZnO NPs formed bulges on *B. cinerea* hyphae surfaces, inhibiting the growth [102]. Quantum dots (QD) and superparamagnetic (SP) NPs were found to interact with fungal hypha. However, the QDs were internalized, likely due to their small 13.5 nm diameter, as opposed to the 100–150 nm diameter SP NPs which seemed to adsorb onto hyphal cell surfaces. QDs were initially (3 h incubation) distributed evenly throughout the cell. Still, at a 6 h incubation time, they were seen to accumulate, indicating some intracellular processing of NPs, although there was also evidence of QD exocytosis [148].

The effect on spores and their germination contributes to the antifungal efficacy of NPs, alongside hyphal deformation. *Penicillium expansum* and its external mycelial colonies treated with ZnO NPs showed damaged conidia and distorted structure, inhibiting conidial germination and development and, ultimately, fungal growth [101]. Another study showed that spores incubated until logarithmic mycelial growth were observed with AgNPs and were shown to have a decreased mycelium growth rate upon germination, with a growth rate depending on the concentrations of AgNPs. However, this was significant in higher concentrations of 5–10 ppm Ag solutions, with a much smaller difference in the <2.5 ppm range [149]. N, F co-doped TiO_2_ NPs exposure inhibited spore germination in a dose-dependent manner. It resulted in damaged mycelia showing ROS production around its surface, unlike the NP-treated samples in the dark or non-NP-treated samples in the light [99]. *F. graminearum* spores suffered damage upon exposure to NPs that collapsed, wrinkled, and distorted structures, giving them a rough surface. The MFC treatment showed more excessive damage than the MIC treatment, but both had apparent morphological destruction of the two types of spores produced by the fungus (macro- and microconidia) [106]. Other NPs, such as AgNPs, reduced the size and septation number of micro- and macroconidia [146].

AgNPs exposure decreased mycelial growth in five fungal species but not in *Mortierella alpina*, which had its growth significantly stimulated through exposure at all tested concentrations. The authors suggest that the known production of polyunsaturated fatty acids by *M. alpina* may act in a chelating fashion, as toxicity from metals often involves a mechanism of lipid peroxidation through the production of free radicals [150,151]. Conidiophore (a branch that produces spores) survival varied amongst the remaining five species and depended on the concentration of NPs and conidiophore location in the mycelium [150]. Despite how this shows that spore survival and germination can be based on certain conditions, generally, spores suffer a change in morphology upon NPs exposure, appearing damaged and shriveled, often coated in NPs [63,69,96]. NPs treatment typically reduces the germination rate, and asexual reproduction can be halted altogether [88,95,114].

There are various theories on how NPs affect spores and what matters in the formulation. Spores possess an overall negative charge on their surface due to the presence of hydrophobins, or low molecular mass proteins secreted by fungi with the ability to self-assemble into amphipathic layers [152]. Nonetheless, negatively charged polymer NPs of comparable sizes were bound more efficiently than positively charged ones, and the increasing positive charge did not enhance attachment or adsorption. This behavior persisted in the ion-free buffer, removing the potential impact of ions; however, the composition of the NPs matters, e.g., SiO_2_ NPs readily coat the hydrophobin layer, whereas CeO_2_ NPs agglomerated in buffer and adsorbed as clusters [116].

Size is necessary, with smaller NPs coating spores more readily. The small NPs experience enhanced binding as there are more surface points since they fit better into conidial surfaces [116]. GO-Ag nanosheets were found to wrap around spore cell walls inhibiting transport and nutrient absorption. Fungal spores mediated GO reduction in oxygen-containing functional groups, likely responsible for GO-AgNPs efficacy. Increasing GO-AgNPs or AgNPs concentration decreased the germination rate of spores [63]. Other studies using metal oxides also found that exposure inhibited sporulation [69,101]. For example, AgNPs reduced spore germination, and germination was halted when formulated biogenically, involving the incorporation of NPs of *Trichoderma viride* metabolites. It is hypothesized that direct interaction with fungi can inhibit conidiospore germination and sporulation [114]. NPs associate with spores immediately upon incubation but do not affect colloidal stability. Kinetic analysis has revealed that NPs coating occurs rapidly (under 30 s), and pH and temperature ranges from 4–8 and 4–55 °C, respectively, did not impact the coating [116].

Contrarily, one study found that NPs coating does not impact spore vitality and reduces its sensitivity to defensins such as human neutrophil peptide 1 and human β-defensin 3, meaning that fungal survival was facilitated by circumventing components of the innate immune system [116]. This could be due to activated defense mechanisms. For example, increased melanin levels have been found in hyphae after exposure to NPs [105]. Melanins are dark polymers that function in fungi to protect the organism from external stresses. Melanin is associated with virulence, protection, pathogenicity, and survival. It can bind antifungal drugs, reduce their effects, and trigger the host complement system [153].

### 9.5. Effect on Biofilm Formation

Fungal biofilms are colonies of sessile cells embedded on a surface. Fungal cells capable of biofilm formation exhibit an increased resistance when in their biofilm morphology. The exudation of extracellular polymeric substances (EPS), increased expression of resistance genes, and altered growth rate contributes to virulence and drug tolerance. Consequently, biofilms are a major clinical concern, particularly in *C. albicans* [154]. Some fungal species, such as *C. albicans,* can form more mature biofilms containing extracellular matrix (ECM) with improved structural integrity than other species, such as *S. cerevisiae.* However, it is not morphology specific, and both yeast and filamentous cells are capable of biofilm development [133].

NPs exposure has been shown to inhibit the formation of biofilm [81,137,155]. Hyphae development is crucial for biofilm formation and adherence, which is required for pathogenesis and colonization. NPs inhibit filamentation, leading to an absence of hyphae and prevention of biofilm development. Cell wall disruption is the driving force behind this inhibition. NPs can also impact pre-formed biofilms and deposit onto EPS, which are essential for structural integrity [137]. It is not unexpected that biofilms would interact with NPs, as it has been shown that *Candida* biofilms may adsorb cations of various metal species onto the ECM, which facilitates resistance. Biofilms are 65 times more tolerant to metal exposure than planktonic cultures. Heavy metals Cu^2+^ and Ni^2+^ form metal-chelator precipitate on ECM, proving that adsorption occurs for the tolerance [156].

Using Ag-doped hydroxyapatite NPs against *C. albicans* biofilms decreased biomass values in the biofilms, showing an impact on ECM production. However, this impact did not remain in the colony-forming units (CFU) count, indicating recovery. Additionally, sub-inhibitory NPs concentrations increased biofilm formation, perhaps due to gene upregulation related to virulence factors [68]. AgNPs exposure was able to reduce *C. albicans* biofilm biomass and CFU number at both intermediate (24 h) and mature (48 h) development phases [157] but demonstrated higher efficacy in biomass reduction when applied to adhered cells vs. pre-formed biofilms, where the incubation time is approximately 2 h, and 48 h, respectively [57].

A study encasing AmB in poly(lactic-co-glycolic acid) NPs found significant *C. albicans* biofilm destruction when the treatment was combined with 15 min of 42 kHz of ultrasound irradiation compared to AmB or ultrasound alone. The treatment reduced biomass and activities of proteinases and phospholipases (known to be virulence factors) and significantly changed biofilm morphology. The same test was done on a catheter biofilm using a rat model, and after seven days of treatment, the biofilm on the catheter surface was eradicated. After treatment, biofilms appeared to have a reduced thickness, with channels and pores throughout. COMSTAT analysis showed that the different treatments had significantly altered thickness, diffusion distance, textural entropy, and areal porosity. Regardless of the use of ultrasound, NPs treatment shortened mycelia and reduced ECM on catheters. Still, after applying ultrasound which causes biofilm dispersal, there was a complete biofilm inhibition [136].

Contrarily, another study found that AgNPs treatment induced EPS-rich biofilm formation and hypothesized that this might be due to the known release of sugar which leads to an environment appropriate for the biofilm formation [55,65,133]. However, AgNPs would adhere to the biofilm and cellular surfaces, ultimately leading to cell damage and growth arrest [65].

### 9.6. ROS Generation

ROS are a family of oxidants often formed as a metabolic by-product and include compounds such as hydrogen peroxide, superoxide anions, and hydroxyl radicals [158]. Mammalian cells in non-viable environmental conditions produce excessive levels of ROS, which cause cellular injury and ultimately end in the arrest of cell growth or cell death [159]. GSH is a thiol in yeast and filamentous fungi crucial for cell function and stress response. GSH has anti-oxidizing properties and reacts with ROS to protect the cell. GSH oligomers termed “phytochelatins” are involved in heavy metal detoxification in fungi through sequestering metal ions or the production of ROS [160].

One of the main antimicrobial characteristics of NPs is attributed to the generation of ROS. For instance, ZnO NPs generate ROS when UV light radiates and oxidizes GSH to form a disulfide from the thiol groups, making glutathione disulfide (GSSG) [103]. Many other NPs induce ROS generation, and activity is often tested by adding a ROS scavenger and observing if NPs effects can be reversed [63,70,103,105,114,140]. SiO_2_ NPs, Al_2_O_3_ NPs, Mn_3_O_4_ NPs, and SnO_2_ NPs generated intracellular ROS that reduced metabolic activity and cell viability. This effect was reversible primarily by adding a free radical scavenger, the ascorbic acid [97]. Another study used the nitroblue tetrazolium reduction assay to detect superoxide radicals in *Alternaria brassicicola*. It showed an accumulation of formazan in AgNP-treated fungal mycelia, indicating that the exhibited structural abnormalities are due to the activity of superoxide radicals [105]. It is hypothesized that structural defects create something termed “disordered sites,” which ultimately result in a change in electronic distribution that, through electron-hole reactions, can generate hydroxyl radicals and superoxide anions [68]. ZnO NPs decreased *C. albicans* viability dose-dependently. When histidine, a known quencher of ROS such as singlet oxygen and hydroxyl molecules [161], was added to the incubation at a 5 mM concentration, the antifungal effect was almost entirely undone. Electron paramagnetic resonance provided further evidence of a ROS-mediated decrease in cell survival. It showed that a reduced *C. albicans* produced ROS upon histidine addition [162].

Decreases in membrane permeability lead to intracellular ROS generation [61]. The increase in lipid peroxidation is hypothesized to be due to ROS production, as this is a known mechanism [105,106,163]. However, a decrease in intracellular ROS also impacts. AgNPs cause a reduction in the secretion of aflatoxin B1 (AFB1), a poisonous carcinogen and mutagen, in *Aspergillus flavus*. When this fungus is cultured, AFB1 is increasingly released, but a sub-inhibitory treatment of AgNPs reduced AFB1 from 66.23 ng/mL when untreated to 28.96 ng/mL with treatment. Simultaneously, superoxide is released from fungal mycelia. It is proposed that the released superoxide causes a reduction in intracellular ROS levels, which triggers suppression of the aflatoxin biosynthesis [89].

ROS release can play a role in antifungal effects, but this is not always the case. One study found that exposure of AuNPs to *C. albicans* did not alter ROS production and hypothesized that the NPs damage and cell death occurring post-exposure must not be from ROS influence. Furthermore, antioxidant treatment did not protect cells from apoptotic processes [164]. Another study found that *C. albicans* experienced an increase in ROS production during the start of culture, but *S. cerevisiae* did not. AgNPs were exposed to *S. cerevisiae* wild type and mutants containing deletions of stress response genes. The AgNPs inhibitory effect was comparable amongst all strains, meaning ROS did not strongly impact the lack of growth [115]. A third study found that the level of superoxide in *S. cerevisiae* cells decreases upon AgNPs exposure, potentially because Ag ions are quenching the ROS [165,166].

There may also be only certain types of radicals whose production is induced. QDs and SP NPs significantly increased superoxides after incubation. Still, QD incubation showed a significant decrease in H_2_O_2_ production, and the H_2_DCFDA signal (representative of ROS accumulation) was not increased in either sample. Therefore only the superoxide anion was raised, and other ROS were unaffected or decreased [148]. This may be due to the materials, as the QDs were made of a CdSe/ZnS core, and the SP NPs were made of SiO_2_, which has been shown in plants to behave protectively and lower ROS [91]. N, F co-doped TiO_2_ NPs generate ROS under visible light around the fungal cell, damaging the cell wall and leading to death. When *F. oxysporum* inoculated with TiO_2_ NPs is grown under visible light, fungal colony growth is halted, whereas it usually progresses in darkness. Under the same conditions with *p*-benzoquinone, a superoxide radical scavenger, and EDTA, an electron-hole scavenger, growth inhibition still occurred, meaning that superoxides and electron holes were not involved in the process [99].

### 9.7. ROS Impact on Mitochondria

One of the most critical ways ROS can affect the fungal cell is through the mitochondria. AgNPs treatment decreased mitochondrial inner membrane potential (ΔΨm) in *C. albicans*, comparable to the positive control using H_2_O_2_. However, the decrease was minimal when cells were treated with AgNPs and thiourea (a hydroxyl radical scavenger). This was tested using DiOC_6_(3) and JC-1 dyes, and both confirmed that AgNPs decreased ΔΨm in a magnitude comparable to H_2_O_2_. At the same time, adding thiourea removed this effect, indicating that preventing hydroxyl radical formation maintains mitochondrial membrane balance, potentially containing an apoptosis [140]. AuNPs caused a mitochondrial increase in Ca^2+^ levels and mitochondrial dysfunction, and as a result, mitochondrial mass in *C. albicans* cells was increased [164]. Ca^2+^ excess in mitochondria plays a part in apoptosis as it can open channels, changing permeability and thus the mitochondrial mass. The homeostasis disturbance leads to reduced mitochondrial membrane potential and cell death by apoptosis. AuNPs exposure also increased cytosolic cytochrome c levels and decreased cytochrome c levels in *C. albicans* mitochondria, making cells no longer able to partake in the electron transport [164].

Cytochrome c resides in the mitochondrial membrane, transferring electrons in the electron transport chain. When the outer mitochondrial membrane experiences a change in permeability (for example, if damage causes swelling to the high surface area of inner cristae, this can damage the outer membrane), cytochrome c will release into the cytosol during early apoptotic phases, triggering a signaling cascade. This has been proven in mammalian cells, and the process is similar in fungi, although it has been found that in yeast, loss of cytochrome c is not necessarily required for programmed cell death [167]. Additionally, the signaling cascade contains caspases that are not in fungi. However, similar compounds termed “metacaspases” exist and were found to be increased upon AgNPs exposure. The addition of thiourea decreased metacaspase levels indicating that AgNPs induce apoptosis with an increase in metacaspases. However, oxidative stress is involved, and once removed, apoptosis is reduced [140]. AuNPs have also increased the metacaspase activation [164].

Flow cytometry has demonstrated mitochondrial membrane potential reduction along with the mitochondrial release of cytochrome C upon AgNPs treatment. Fluorescence microscopy showed apoptosis through nuclear fragmentation, metacaspase activation, and phosphatidylserine externalization. Additionally, ROS levels were increased. The AgNPs accumulate intracellularly in *C. albicans*, increasing radical hydroxyl levels, a factor in yeast apoptosis. Adding thiourea decreases apoptosis mediated by mitochondrial dysfunction, suggesting that the hydroxyl radical is critical for apoptosis [140].

### 9.8. Gene Regulation and Protein Levels

Gene expression and protein levels can be altered upon NPs exposure. As discussed previously, gene up- and downregulation can confer resistance to antifungals in fungal species. So, these changes would be expected upon interaction with a compound with antimycotic activity.

Multiple studies investigated the effect of NPs on the *S. cerevisiae* genome. Horstmann et al. found that exposure to AgNPs altered gene expression in 1845 genes: 1077 upregulated and 768 downregulated [165]. Of 1077 genes, 651 were involved in nitrogen compound metabolism. Other upregulated categories included ribosome biogenesis, rRNA processing, translation, and translational initiation. *DBP2* and *FAF1* genes involved in rRNA processing were upregulated 7.9-fold and 7.2-fold, respectively, and the most upregulated gene was found to be *CTR1*, responsible for copper uptake in low copper environments. *S. cerevisiae* does contain metallothioneins that uptake Cu in the form of Cu(I); thus, it is possible that Ag can trigger Cu-related mechanisms [168]. The majority (25.3%) of downregulated genes were involved in the single-organism metabolic process. While many of the most downregulated genes had an unknown function, other top categories included lipid metabolic processes (such as genes involved in ergosterol synthesis: *ERG3*, *ERG5*, *ERG6*, *ERG11*, *ERG25*, *ERG28*), transmembrane transporters, and mitochondrial cellular respiration [168]. *AAC3* and *MPC3*, involved in the transport of ADP/ATP and pyruvate in the mitochondria, were downregulated, along with *ACO1*, *CIT3*, and *ISF1,* which are involved in mitochondrial respiration as well. Other downregulated genes included those regulating oxidative stress responses (*GRX6*, *TSA2*, *VHR1*, *ZTA1*), regulating osmotic stress response (*CIN5*, *NRG2*), and involved in spore cell wall formation (*GIP1*, *GSC2*, *OSW2*, *SPO73*), and involved in cell wall mannoprotein production (*TIP1*, *TIR1*, *TIR2*, *TIR3*, *TIR4*, *DAN1*), which included a protein that experienced a 174-fold downregulation (*DAN1*). In conclusion, the authors hypothesized that sublethal AgNPs levels stimulate ribosome biogenesis in the nucleolus and inhibit mannoprotein and glycan turnover, negatively impacting cell wall integrity. The decrease in sugar transporters and plasma membrane destabilization may also impact cytosol sugar levels. Finally, AgNPs damaged mitochondria, halting proper respiratory chain function, generating ROS, and inducing apoptosis [165].

Babele et al. investigated gene expression in *S. cerevisiae* upon exposure to the ZnO NPs treatment [104]. They found upregulation of the chitin synthesis genes *CHS1*, *CHS3*, and *CHS5*, indicating damage to the cell wall. Further analysis was done on genes involved in cell wall integrity, redox homeostasis, and the HOG signaling pathway (High Osmolarity Glycerol, involved in cell response to hyperosmotic stress). The HOG pathway consists of a MAP kinase cascade and works in conjunction with the cell wall integrity (CWI) pathway to respond to the cell stress [169,170]. When treated with ZnO NPs, growth was inhibited in yeast with the following null mutations: ROM1, MEK MKK1, MEK MKK2, MEKK BCK1, MAPK SLT2, RLM1, and GAS1, suggesting that the CWI pathway is essential for ZnO NPs tolerance and that there is involvement with MAP kinase [104].

To investigate lipid metabolism and homeostasis, fatty acid synthase genes *FAS1* and *FAS2* were examined, and it was found that their proteins accumulated in cytosol upon ZnO treatment, unlike the untreated control, where the proteins were evenly dispersed. Similar trends of accumulation in foci were observed when studying mitochondria and HSP1, a heat-shock protein necessary for stress management. Mitochondrial PSD1 was found localized in the cytosol, supporting the finding of mitochondrial dysfunction while also indicating lipid mechanism defect. So, ZnO NP-treated cells experienced damage to both the mitochondria and endoplasmic reticulum (ER), further evidenced by the upregulation of the *IRE1* gene and an accumulation of activated HAC1 forms. *IRE1* is a sensor for the unfolded protein response (UPR), an ER stress response related to faulty protein production, and HAC1 is concomitant with UPR activation. The mitochondrial/ER protein complex, ERMES, appeared damaged as both components were affected. The ER is necessary for protein folding, lipid synthesis, and redox homeostasis [171,172,173,174]. Because it has been hypothesized that the ER and cell wall stress responses are intertwined with the UPR and CWI pathway [172], it is not surprising that both pathways are impacted by NP exposure. Finally, treated cells showed an increase in cellular lipid droplets and the presence of ATG8 in vacuoles, indicating the start of autophagy. Ultimately, this study found that ZnO NPs treatment impacted stress mechanisms involving the cell wall, the ER, and the mitochondria, leading to cell death.

There is a possibility that specific genes are regulated not because of the NPs but rather by the released ions. A transcriptomic analysis using AgNPs and Ag ions treatments in *S. cerevisiae* found a ~3% and ~5% change in genome expression, respectively [142]. Most up- and down-regulation in known gene functions at earlier incubation stages (120 min) included heat shock, cell wall proteins, oxidative stress, and metabolism. Later (210 min), incubation affected genes like Ag ion exposure, suggesting AgNPs toxicity evolves to be similar to Ag ion toxicity over time. Targeted genes involved iron retention, cell division, stress, and transporter proteins. The most upregulated genes for both AgNPs and Ag ions were *CUP1-1* and *CUP1-2* (responsible for metallothionein production involved in Cu and Cd resistance, known to be upregulated in response to cytosolic Cu increase), *PHO89* (Na+/Pi cotransporter), and heat shock genes (e.g., *HSP12*, *HSP26*) [142].

*A. brassicicola* was also tested with AgNPs, and its stress enzyme activities (verified by calculating the content of thiobarbituric acid reactive substances) of superoxide dismutase (SOD), catalase, ascorbate peroxidase (APX) and glutathione peroxidase (GPX) decreased [105]. All of these enzymes play a role in protecting the cell from oxidative damage. The same study used reverse transcription polymerase chain reaction (RT-PCR) to examine the different gene expressions between AgNPs made chemically (CSNPs) and AgNPs made using *T. viride* (BSNPs). Both NPs heavily downregulated *ATM*, responsible for transmembrane protein synthesis as a part of intracellular redox regulation, indicating issues with this homeostasis. Both NPs also heavily upregulated *SLT2*, a gene involved in a signal cascade for cell wall maintenance, indicating cell wall damage. Interestingly, genes *NPS2*, *NIK*, and *AMK* experienced downregulation upon BSNPs exposure and upregulation upon CSNPs exposure [105]. The varied expression of these genes, involved in various tasks such as spore survival, virulence, and cell wall and osmotic homeostasis, show that the fungi can attempt recovery when exposed to CSNPs, while BSNPs exhibit fungicidal behavior.

The different enzymatic levels in five fungal strains isolated from streams without metal pollution vs. with metal pollution upon CuO NPs exposure were studied to investigate how metal pollution can alter the gene expression regulation [141]. Glutathione reductase (GR), responsible for catalyzing GSSG to GSH, and GPX, responsible for catalyzing GSH to GSSG, increased with the CuO NPs exposure [141,173]. Fungi isolated from non-polluted streams had higher GPX and lowered GR activity, especially after prolonged exposure. It is possible that peroxide radicals were not scavenged as thoroughly by fungi from non-polluted streams, so they experienced lower levels of GSH. In comparison, fungi from polluted streams maintained higher GSH levels. SOD activity also increased concomitantly with CuO NPs in all fungi, more severely than other antioxidant enzymes. The level was higher in fungi isolated from metal-polluted streams [141]. It has been previously shown that aquatic fungi exposed to metals including Cu produce compounds such as thiols, small peptides (likely glutathione and phytochelatins), and metallothioneins, the latter of which likely behave as ROS scavengers [174]. Investigating the difference in metal pollution showed that the ionic Cu form is more impactful than its NPs form, perhaps due to bioavailability. Ultimately, metal adaptation in fungi can lead to mechanisms of metal tolerance, mainly dealing with oxidative stress, that can transfer to tolerating NPs exposure [141].

Other tolerance mechanisms include efflux pump production, melanin synthesis, and biofilm formation. This is also reflected in gene regulation. AgNPs exposure downregulated the expression of genes polyketide synthase (*PKS1*) and scytalone dehydratase (*SCD1*), both involved in melanin biosynthesis, by 6.47-fold and 1.8-fold, respectively [88]. *CDR1*, *CYP51A*, *HSP90*, and *ERG11* were significantly downregulated at antifungal concentrations in both fluconazole-resistant and –susceptible *C. albicans* strains when exposed to SeNPs [71,72]. These genes have been linked to resistance mechanisms [175,176,177]. *CDR* genes are responsible for efflux pumps, and *CDR1* is often overexpressed in resistant strains [178], *ALS* (agglutinin-like sequence), *SAP* (secreted aspartyl protease), *PLB* (phospholipase B), and *LIP* (lipase) gene families have all shown involvement in *C. albicans* biofilm formation, along with HWP1 (hyphal wall protein) [178]. HWP1 is a GPI-linked protein also associated explicitly with hyphae. It has been shown to confer adherence and virulence [179]. ZnO NPs decreased *HWP1* gene expression in *C. albicans* isolates [155]. The ALS gene family encodes GPI-linked cell surface glycoproteins. This is important for host cell invasion. *ALS3,* in particular, is upregulated during infection [179]. Sub-inhibitory ZnO NPs exposure downregulated the expression of *ALS1* and *ALS3* in fluconazole-resistant *C. albicans* strains significantly more than fluconazole treatment [74]. Secreted aspartyl proteinases (SAP) are extracellular hydrolytic enzymes coded by 10 *SAP* gene families, known to be produced by many pathogenic *Candida* species, including *C. albicans*. SAP is involved in virulence and is responsible for evading immune response, acquiring cell nutrition, and facilitating invasion. SAP is produced in higher concentrations in more resistant strains [180]. *C. albicans* treated with subinhibitory levels of ZnO NP decreased *SAP1*, *SAP2*, and *SAP3* gene expression 2.44-fold, 5.55-fold, and 2.6-fold, respectively. Nystatin showed similar decreases at 4.16-fold, 3.12-fold, and 4.8-fold, respectively [76].

**Figure 5:**
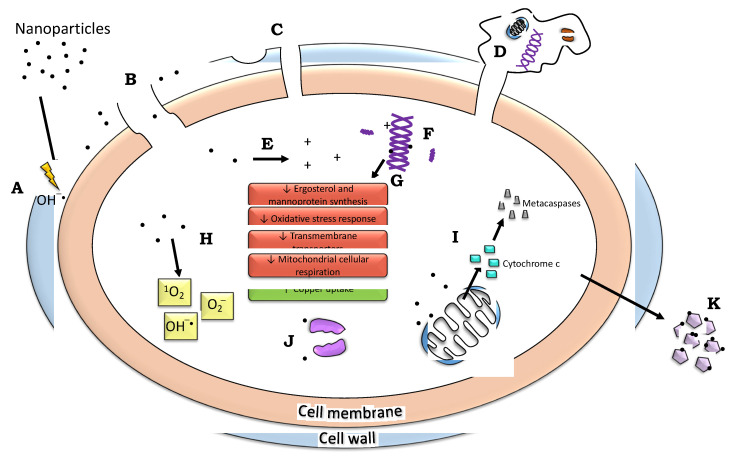
NPs mechanisms at the cellular level that lead to fungal cell damage include (**A**) ROS-inducing lipid peroxidation, (**B**) adsorption embedment and breakage of cell wall and membrane, (**C**) pit and pore formation, (**D**) leakage, releasing DNA and organelles from the cell, (**E**) ion release, (**F**) DNA intercalation, causing condensation and fragmentation, (**G**) gene expression changes, (**H**) ROS generation, (**I**) Mitochondrial release of cytochrome C into the cytosol, increasing metacaspase levels, leading to apoptosis cascade, (**J**) ribosome depolymerization, and (**K**) adsorption onto EPS, inhibiting biofilm formation.

The global damages caused as a result of the exposure of the fungal cell to NPs is pictured in Figure 5.

## 10. Experimental Conditions

The parameters and conditions under which an experiment is done can alter results. Specific NPs formulations may require special considerations during experimentation, such as light exposure or an applied magnetic field. Still, factors such as the media used also change the results and can do so significantly. For example, an experiment tested the effects of AgNPs on *Fusarium culmorum* spores in three different media: nutrient-rich potato dextrose agar (PDA), nutrient-poor PDA, and agar. The nutrient-rich PDA required an incubation time with AgNPs of 24 h for antimicrobial activity and found AgNPs concentration variation to be insignificant. Nutrient-poor PDA found AgNPs significantly reduced mycelial growth regardless of incubation time or concentration. In agar, a low 0.12 ppm concentration increased mycelial growth, while 1.25 ppm was comparable to the control, and only 2.5 ppm slightly decreased. Additionally, mycelium in the agar media did not form spores [149]. Compared to malt extract agar (MEA) or corn meal agar (CMA), PDA found more promising results, with 90–100% inhibition of mycelial growth rate across 18 fungal species [87].

This effect of media is unsurprising as it is known that fungi will change metabolism depending on external stresses. If fungi are carbon deprived, this triggers GSH activity and adapts the organism to the oxidative stress [159]. *C. albicans* reacts to environmental conditions, which can trigger morphogenesis. The addition of serum to the culture, change in pH, or change in incubation temperature can all promote the transition of *C. albicans* yeast cells into filamentous forms [161]. Growing yeast cells in <0.1% glucose changes cell wall structure, decreasing chitin and β-glucan count and decreasing mannan chain length [181]. Compounds in complex media (e.g., glucose) can also behave as reducing agents, creating NPs from metal salts. As a result, during fungal use for NPs formation, the media within which the fungi are cultured can affect NPs characteristics [107].

Incubation time is another factor that changes results. Fungal growth during the logarithmic growth phase post-inoculation is impacted by both the growth medium used and incubation time (with and without NPs) [149]. It is also worth noting that there is no standardized incubation time to compare all experimental assays, and almost every study testing any mechanism (e.g., cell wall interaction or mycelial growth inhibition) across multiple time points will report varying results, usually either increased efficacy overtime or recovery [81,114,135,141,142,145].

Fungal species used in experiments can also produce different results for various reasons. Some strains acquire resistance and thus survive using mechanisms other than their non-resistant counterparts, many of which are discussed in this review. If NPs target any of these mechanisms, the outcome differs based on strain. There is also a difference between pathogenic and non-pathogenic fungi, and increased AgNPs efficacy against pathogenic fungi indicates that some AgNPs formulations function by targeting a pathogenic pathway [115]. Lastly, fungal growth patterns can also alter results; fungi that grow in a more dense matter may be more sensitive to NPs due to the potential of increased exposure [102].

## 11. Potential Applications of the Use of Metallic NPs with Antifungal Activity

### 11.1. Agriculture

One of the main challenges of farmers is to control the production of their produces. Phytopathogenic fungi decimate significant farming areas, and their control burden agriculture. Although fungi are controlled with synthetic chemical products, their indiscriminate use creates environmental problems, such as pollution, water contamination, reduction in pollinators, the disappearance of species, toxicity to humans and animals, etc. More challenging is the development of fungal resistance against these chemical products.

Moreover, fungal diseases threaten biodiversity and agricultural practices. Fungal infection can decrease root and shoot lengths and root and shoots dry weight [88]. NPs could be a potential alternative to pesticides, allowing the control of fungal pathogens in a comparable or superior way to current chemical methods. Many studies have shown NP’s antimycotic activity against fungi that cause crop diseases [79,86,91,99,100,140,145,146,148,182]. The application of AgNPs in a greenhouse experiment using *Triticum aestivum* infected with *Bipolaris sorokiniana* increased root length, shoot length, and shoot dry weight by 11.55%, 30.67%, and 60.83%, respectively, when compared to untreated plants [88].

Interestingly, this study also found that applying AgNPs increased lignification, a known factor in plant development and disease resistance [183], to an intensity similar to healthy control. Infected and untreated plant lignification was barely visible through histochemical staining. SiO_2_ NPs used to treat *Rhizoctonia solani* were able to alleviate symptoms of infection in seedlings. The NPs exposure increased levels of photosynthetic pigments (chlorophylls and carotenoids), reduced lipid peroxidation, enhanced root and shoot lengths and fresh and dry weight of seedlings, increased salicylic acid levels (leads to the defense of lignification), induced phenolic and flavonoid antioxidant activity, and upregulated genes *POD*, *SOD*, *APX*, *CAT*, and *PPO* to manage oxidative stress. Therefore SiO_2_ NPs were able to prevent the harm of *R. solani* infection and help seedlings maintain homeostasis [91].

### 11.2. Dentistry

One of the applications of metallic NPs with antifungal activity is in oral diseases. One of the most studied fungi affecting the oral cavity is the opportunistic pathogen *C. albicans*. This yeast is relevant in different patients with specific treatments that weaken the immune response, e.g., leukemia, malnutrition, immunosuppression, radiation, and chemotherapy [184]. These patients have shown growth of *C*. *albicans* in denture prosthetics with a concomitant resistance against conventional antifungal agents.

One study showed the development of a polymeric agent consisting of a mixture of AgBr-NPs, quaternary ammonium salt polymer 4-vinyl pyridinium (NPVP), mixed with a denture resin such as polymethylmethacrylate (PMMA) showed antifungal activity against *C*. *albicans*. The AgBr-NPs used had an average size of 30 nm with a MIC of 250 µg/mL [185].

### 11.3. Biomedical

Using cotton fibers covered by metallic NPs is an attractive application for topical infections or burns. For example, anti-*Candida* activity was achieved when the fibers were covered with CuNPs. The antifungal activity remained even after washing or ironing for 6 months [186].

Another composite that showed antifungal activity against *C*. *albicans* was ZnONPs with a 30 nm diameter coated with chitosan-linoleic acid. This nanocomposite showed MICs of 32 mg/mL vs. 8 mg/mL measured for fluconazole [187].

Lastly, metallic prostheses fabricated with titanium are susceptible to being covered by the synthesis of *C*. *albicans* biofilms [188]. When titanium was coated with poly(dimethyl siloxane) (PDMS), a strong antifungal activity was measured with a decrease in the *C*. *albicans* biofilm biosynthesis [189].

### 11.4. Industrial

The food industry is another attractive field for developing metallic NPs for fungal control. For instance, aflatoxins are produced by filamentous fungi such as the genus *Aspergillus*. These compounds are toxins with known mutagenic and allergenic activities with severe clinical implications [190]. The use of poly(lactic acid) as a matrix covered with ZnO NPs showed an inhibitory effect against *A. flavus* and *A. parasiticus* [191].

Another potential application of ZnO NPs is preventing spore germination on air filters. In this way, the dissemination of fungi could be restricted. This is critical in food manufacturing facilities where the air is filtered for trapping microorganisms, dust, and other particles.

**Figure 6:**
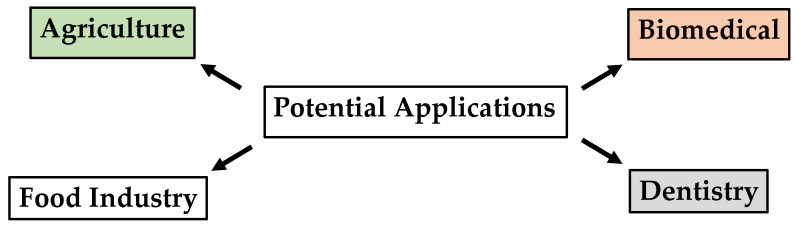
Potential application of NPs with antifungal activity to different industries.

A summary of the potential applications of the NPS is presented in Figure 6.

## 12. Conclusions

There is a lack of knowledge pertaining to the mechanism of action when it comes to the antifungal activity of NPs. NP-mediated fungal cell toxicity occurs through many mechanisms, including cell wall damage and change (such as ergosterol synthesis inhibition), gene regulation, interaction with reproductive structures and hyphae, and ROS production, causing cascades of damages such as lipid peroxidation and mitochondrial damage. This multi-faceted attack makes NPs an avenue worth investigating as potential future antifungal treatments. Due to the multitude of factors involved in NPs formation, such as shape, size, and composition, combined with the variety of fungal strains that undergo testing, it is challenging to compare and contrast experiments and pinpoint what factors are more crucial than others. However, it is apparent in the literature that NPs exhibit a promising antifungal effect that can be enhanced or customized through formulation. These NPs could be helpful in a clinical setting, either in medical applications such as functionalized with known antifungals or on medical devices and surfaces.

Further studies are necessary to develop metallic NPs for different applications as their use so far is very limited. In the case of their applications as antifungal in packaging should consider more toxic studies because of their contact with meals, fruits, and vegetables. Additionally, the available techniques used to determine the level of metallic NPs are deficient when studying NPs that interact with food.

Also, the impact of metallic NPs on the environment should be considered, including their effects on aquatic life and the health risks associated with their use.

## Figures and Tables

**Table 1:** Activities of NPs tested on different fungal species expressed in MIC_100_ (µg/mL).

Type of NP	Shape	Size (nm)	Organism(s) Tested	MIC_100_ (µg/mL)	Reference
Ag	Spherical	3	*Saccharomyces cerevisiae* (KCTC 7296) *Trichosporon beigelii* (KCTC 7707) *Candida albicans* (ATCC 90028)	2	[55]
	Spherical	1–21	*Candida albicans* *Candida glabrata* *Candida parapsilosis* *Candida tropicalis* *Fusarium solani* *Fusarium moniliforme* *Fusarium oxysporum* *Aspergillus flavus* *Aspergillus fumigatus* *Aspergillus terrus* *Sporothrix schenckii* *Cryptococcus neoformans*	0.25 0.125 0.25 0.125 1 2 4 1 2 2 0.25 0.25	[56]
	Spherical	5	*Candida albicans* (324LA/84) *Candida glabrata* (ATCC 90030) *Candida albicans* (ATCC 10321) *Candida glabrata* (D1)	0.4–0.8 0.4–0.8 0.8–1.6 1.6–3.3	[57]
	Spherical	7	*Aspergillus flavus* *Aspergillus fumigatus*	100 100	[58]
	Spherical	25	*Candida albicans* (I) *Candida albicans* (II) *Candida parapsilosis* *Candida tropicalis*	0.42 0.21 1.690 0.840	[59]
	Spherical	5–20	*Trichosporon asahii* (CBS2479) *Trichosporon asahii* (BZ701) *Trichosporon asahii* (BZ702) *Trichosporon asahii* (BZ703) *Trichosporon asahii* (BZ704) *Trichosporon asahii* (BZ705) *Trichosporon asahii* (BZ705R) *Trichosporon asahii* (BZ901) *Trichosporon asahii* (BZ902) *Trichosporon asahii* (BZ121) *Trichosporon asahii* (BZ122) *Trichosporon asahii* (BZ123) *Trichosporon asahii* (BZ124) *Trichosporon asahii* (BZ125) *Trichosporon asahii* (CBS8904) *Trichosporon asahii* (CBS7137) *Trichosporon asahii* (CBS8520)	0.50 0.67 0.50 1.00 0.67 0.50 0.50 0.67 1.00 0.83 0.67 0.50 0.67 0.83 0.50 0.67 0.67	[60]
	Spherical	15–25	*Candida albicans* (ATCC 10231) *Candida albicans* (ATCC 90028) *Candida glabrata* (ATCC 90030) *Candida parapsilosis* (ATCC 22019)	1.56 6.25 3.12 6.25	[61]
	Spherical	25	*Candida albicans* (I) *Candida albicans* (II) *Candida parapsilosis* *Candida tropicalis*	27 27 27 27	[59]
	Spherical to polyhedric	73.72	*Trichophyton rubrum* (*n* = 8) *Trichophyton rubrum* (ATCC MYA 4438)	0.5–2.5 <0.25	[62]
	Spherical	76.14	*Trichophyton rubrum* (*n* = 8) *Trichophyton rubrum* (ATCC MYA 4438)	>7.5 >7.5	[62]
	Spherical	100.6	*Trichophyton rubrum* (*n* = 8) *Trichophyton rubrum* (ATCC MYA 4438)	0.5–5 0.5	[62]
	NR	NR	*Fusarium graminearum*	4.68	[63]
	NR	NR	*Candida albicans* (I) *Candida albicans* (II) *Candida parapsilosis* *Candida tropicalis*	0.42 0.21 1.69 0.84	[59]
	NR	NR	*Candida albicans* (I) *Candida albicans* (II) *Candida parapsilosis* *Candida tropicalis*	0.052 0.1 0.84 0.42	[59]
	NR	NR	*Candida albicans* (I) *Candida albicans* (II) *Candida parapsilosis* *Candida tropicalis*	3.38 3.38 3.38 3.38	[59]
	NR	20–25	*Aspergillus niger* *Candida albicans* *Cryptococcus neoformans*	25 6 3	[64]
	NR	NR	*Fusarium graminearum*	12.5	[63]
Ag/ZnO	Spherical	7/477	*Aspergillus flavus* *Aspergillus fumigatus*	50/10 50/10	[58]
qAg	Spherical	2–3	*Candida albicans*	0.07	[65]
Cu	Spherical	10–40	*Candida albicans* (ATCC 10231) *Candida albicans* (Clinical strain C) *Candida albicans* (Clinical strain E)	129.7 1037.5 518.8	[66]
	Wires	20–30 µm, 30–60 nm diameter	*Candida albicans* (ATCC 10231) *Candida albicans* (Clinical strain C) *Candida albicans* (Clinical strain E)	260.3 260.3 260.3	[66]
γ-Fe_2_O_3_/Ag	NR	20–40 (Ag) + 5 (γ-Fe_2_O_3_)	*Candida albicans* (I) *Candida albicans* (II) *Candida tropicalis* (5) *Candida parapsilosis* (6)	1.9 1.9 31.3 31.3	[67]
Fe_3_O_4_/Ag	NR	~5 (Ag) + ~70 (Fe_3_O_4_)	*Candida albicans* (I) *Candida albicans* (II) *Candida tropicalis* (5) *Candida parapsilosis* (6)	1.9 1.9 3.9 7.8	[67]
GO/Ag	Spherical	10–35	*Fusarium graminearum*	9.37	[63]
HA/Ag	Rod/Spherical	12–27	*Candida albicans*	62.5	[68]
MgO 500 °C calcination	Flaked layers	52 ± 18	*Colletotrichum gloeosporioides* (from papaya) *Colletotrichum gloeosporioides* (from avocado)	156 312	[69]
MgO 1000 °C calcination	Flaked layers	96 ± 33	*Colletotrichum gloeosporioides* (from papaya) *Colletotrichum gloeosporioides* (from avocado)	312 312	[69]
Pd	Spherical	9 ± 3.9	*Candida albicans* (ATCC 10231) *Aspergillus niger*	212.5 200	[70]
Se	Spherical	80–200	*Candida albicans* (ATCC 76615) *Candida albicans* (ATCC 10231)	100 70	[71]
Se	Spherical	37–46	*Aspergillus fumigatus* (TIMML-025) *Aspergillus fumigatus* (TIMML-050) *Aspergillus fumigatus* (TIMML-379)	0.5 0.5 1	[72]
Silica/AmB	NR	5	*Candida albicans* *Candida krusei* *Candida parapsilosis* *Candida glabrata* *Candida tropicalis*	100 1000 1000–2000 300 100	[73]
TiO_2_/Ag + NaHB_4_	NR	250–300	*Aspergillus niger* *Candida albicans* *Cryptococcus neoformans*	25 12.5 12.5	[64]
TiO_2_/Ag + UV	NR	250–300	*Aspergillus niger* *Candida albicans* *Cryptococcus neoformans*	12.5 6 3	[64]
ZnO	Spherical	20–40	*Candida albicans* (*n* = 125)	0.2–296	[74]
	Spherical	477	*Aspergillus flavus* *Aspergillus fumigatus*	20 20	[58]
ZnO 500 °C calcination	Spherical + cylindrical	51 ± 13	*Colletotrichum gloeosporioides* (from papaya) *Colletotrichum gloeosporioides* (from avocado)	156 312	[69]
ZnO 1000 °C calcination	Hexagonal bars	53 ± 17	*Colletotrichum gloeosporioides* (from papaya) *Colletotrichum gloeosporioides* (from avocado)	156 312	[69]
ZnO/Mg(OH)_2_ 25 °C synthesis	Flakes + bars	54 ± 17	*Colletotrichum gloeosporioides* (from papaya) *Colletotrichum gloeosporioides* (from avocado)	156 312	[69]
ZnO NP 25 °C synthesis	Hexagonal bars + ovoidal	63 ± 18	*Colletotrichum gloeosporioides* (from papaya) *Colletotrichum gloeosporioides* (from avocado)	312 312	[69]
ZnO/Mg(OH)_2_ 70 °C synthesis	Flakes + bars	71 ± 22	*Colletotrichum gloeosporioides* (from papaya) *Colletotrichum gloeosporioides* (from avocado)	312 312	[69]
ZnO 25 °C synthesis, hydrothermal	Prisms with pyramidal ends	77 ± 31	*Colletotrichum gloeosporioides* (from papaya) *Colletotrichum gloeosporioides* (from avocado)	312 312	[69]
ZnO/Mg(OH)_2_ NP 25 °C synthesis, hydrothermal 160 °C	Flakes + bars	88 ± 30	*Colletotrichum gloeosporioides* (from papaya) *Colletotrichum gloeosporioides* (from avocado)	312 312	[69]
ZnO/Mg(OH)_2_ 70 °C synthesis, hydrothermal 160 °C	Flakes + bars	98 ± 41	*Colletotrichum gloeosporioides* (from papaya) *Colletotrichum gloeosporioides* (from avocado)	312 312	[69]
ZnO/MgO 25 °C synthesis, 500 °C calcination	Flakes	139 ± 49	*Colletotrichum gloeosporioides* (from papaya) *Colletotrichum gloeosporioides* (from avocado)	312 312	[69]
ZnO/MgO 70 °C synthesis, 500 °C calcination	Flakes	161 ± 44	*Colletotrichum gloeosporioides* (from papaya) *Colletotrichum gloeosporioides* (from avocado)	312 312	[69]
ZnO/MgO 70 °C synthesis, 1000 °C calcination	Flakes	219 ± 39	*Colletotrichum gloeosporioides* (from papaya) *Colletotrichum gloeosporioides* (from avocado)	625 625	[69]
ZnO	NR	NR	*Candida albicans* (*n* = 10)	0.02–269	[75]

MIC, minimal inhibitory concentration; qAg, quantum silver; NR, Not reported; HA, hydroxyapatite.

**Table 2:** Activities of NPs tested on different fungal species expressed as MIC_50_ (µg/mL).

Type of NP	Shape	Size (nm)	Organism(s) Tested	MIC_50_ (µg/mL)	Reference
Ag	Spherical	30–50	*Candida albicans* (*n* = 30) *Candida glabrata* (*n* = 30) *Candida tropicalis* (*n* = 30)	4 9 9	[76]
Ag	Cubical	40–50	*Candida albicans* (*n* = 30) *Candida glabrata* (*n* = 30) *Candida tropicalis* (*n* = 30)	1 7 7	[76]
Ag	Wires	250–300	*Candida albicans* (*n* = 30) *Candida glabrata* (*n* = 30) *Candida tropicalis* (*n* = 30)	5 12 11	[76]
Au	Cubical	30–50	*Candida albicans* (*n* = 30) *Candida glabrata* (*n* = 30) *Candida tropicalis* (*n* = 30)	3 10 11	[76]
Au	Spherical	35–50	*Candida albicans* (*n* = 30) *Candida glabrata* (*n* = 30) *Candida tropicalis* (*n* = 30)	8 13 12	[76]
Au	Wires	300–500	*Candida albicans* (*n* = 30) *Candida glabrata* (*n* = 30) *Candida tropicalis* (*n* = 30)	7 15 15	[76]
ZnO	Spherical	20–40	*Candida albicans* (*n* = 125)	8.2	[74]
ZnO	NR	NR	*Candida albicans* (*n* = 10)	5	[75]

MIC, minimal inhibitory concentration; NR, Not reported.

**Table 3:** Activities of NPs tested on different fungal species expressed as MIC_80_ (µg/mL).

Type of NP	Shape	Size (nm)	Organism(s) Tested	MIC_80_ (µg/mL)	Reference
AuNP + SnCl_2_ as reducing agent	Polygonal, almost spherical	5–50	*Candida albicans* *Candida tropicalis* *Candida glabrata*	16 16 16	[77]
AuNP + NaBH_4_ as reducing agent	Spherical	3–20	*Candida albicans* *Candida tropicalis* *Candida glabrata*	4 4 4	[77]

MIC, minimal inhibitory concentration.

**Table 4:** Activities of NPs tested on different fungal species expressed as MIC_90_ (µg/mL).

Type of NP	Shape	Size (nm)	Organism(s) Tested	MIC_90_ (µg/mL)	Reference
LMW Chitosan NP ± 1 mg/mL chitosan	Spherical	174 ± 38.47	*Candida albicans* *Fusarium solani* *Aspergillus niger*	250 1000 NR	[78]
HMW Chitosan NP ± 1 mg/mL chitosan	Spherical	210 ± 24.54	*Candida albicans* *Fusarium solani* *Aspergillus niger*	1000 500 NR	[78]
LMW Chitosan NP ± 2 mg/mL chitosan	Spherical	233 ± 41.38	*Candida albicans* *Fusarium solani* *Aspergillus niger*	857.2 857.2 NR	[78]
HMW Chitosan NP ± 2 mg/mL chitosan	Spherical	263 ± 86.44	*Candida albicans* *Fusarium solani* *Aspergillus niger*	857.2 857.2 1714	[78]
LMW Chitosan NP ± 3 mg/mL chitosan	Spherical	255 ± 42.81	*Candida albicans* *Fusarium solani* *Aspergillus niger*	607.2 1214 NR	[78]
HMW Chitosan NP ± 3 mg/mL chitosan	Spherical	301 ± 72.85	*Candida albicans* *Fusarium solani* *Aspergillus niger*	607.2 1214.3 2428.6	[78]
Ag	Cubical	40–50	*Candida albicans* (*n* = 30) *Candida glabrata* (*n* = 30) *Candida tropicalis* (*n* = 30)	8 30 31	[76]
Ag	Spherical	30–50	*Candida albicans* (*n* = 30) *Candida glabrata* (*n* = 30) *Candida tropicalis* (*n* = 30)	11 37 35	[76]
Ag	Wires	250–300	*Candida albicans* (*n* = 30) *Candida glabrata* (*n* = 30) *Candida tropicalis* (*n* = 30)	21 51 48	[76]
Au	Cubical	30–50	*Candida albicans* (*n* = 30) *Candida glabrata* (*n* = 30) *Candida tropicalis* (*n* = 30)	10 45 46	[76]
Au	Spherical	35–50	*Candida albicans* (*n* = 30) *Candida glabrata* (*n* = 30) *Candida tropicalis* (*n* = 30)	15 47 48	[76]
Au	Wires	300–500	*Candida albicans* (*n* = 30) *Candida glabrata* (*n* = 30) *Candida tropicalis* (*n* = 30)	30 70 73	[76]
ZnO	Spherical	20–40	*Candida albicans* (*n* = 125)	17.76	[74]
ZnO	NR	NR	*Candida albicans* (*n* = 10)	11.3	

MIC, minimal inhibitory concentration; NR, Not reported; LMW, Low-molecular-weight; HMW, High-molecular-weight.

**Table 5:** Activities of NPs tested on different fungal species expressed as the zone of inhibition (mm).

Type of NP	Shape	Size (nm)	Organism(s) Tested	Activity (mm)	Reference
CuO	Spherical	3–30	*Fusarium equiseti* *Fusarium oxysporum* *Fusarium culmorum*	25 20 19	[79]
Pd	Spherical	200	*Colletotrichum gloeosporioides*	Day 2: 3.6 Day 4: 1.6	[80]
			*Fusarium oxysporum*	Day 2: 12.2 Day 4: 10.9	
Pd	Spherical	220	*Colletotrichum gloeosporioides*	Day 2: 7.9 Day 4: 6.3	[80]
			*Fusarium oxysporum*	Day 2: 5.1 Day 4: 4.7	
Pd	Spherical	250	*Colletotrichum gloeosporioides*	Day 2: 2.4 Day 4: 0.7	[80]
			*Fusarium oxysporum*	Day 2: 10.4 Day 4: 9.6	
Pd	Spherical	350	*Fusarium oxysporum*	Day 2: 1.5 Day 4: 1.3	[80]
Pd	Spherical	550	*Fusarium oxysporum*	Day 2: 3.8 Day 4: 3.3	[80]
Nystatin/MgO/CuO	Spherical	8000–10000	*Candida albicans* (AH201) *Candida albicans* (AH267)	24.5 ± 1.7 14.3 ± 1.2	[81]
Nystatin	Spherical	8000–10000	*Candida albicans* (AH201) *Candida albicans* (AH267)	0.41 ± 0.23 0.5 ± 0.21	[81]
MgO/CuO	Spherical	8000–10000	*Candida albicans* (AH201) *Candida albicans* (AH267)	19.2 ± 1.6 1.3 ± 0.61	[81]
TiO_2_/BPE B	NR	NR	*Candida albicans* (ATCC 14053)	11.2 ± 0.02	[82]
TiO_2_/BPE C	NR	NR	*Candida albicans* (ATCC 14053)	15.9 ± 0.04	[82]
TiO_2_/BPE D	NR	NR	*Candida albicans* (ATCC 14053)	13.5 ± 0.04	[82]
TiO_2_/BPE E	NR	NR	*Candida albicans* (ATCC 14053)	14.6 ± 0.01	[82]
TiO_2_/BPE B	NR	NR	*Penicillum chrysogenum* (MTCC 5108)	10.2 ± 0.05	[82]
TiO_2_/BPE C	NR	NR	*Penicillum chrysogenum* (MTCC 5108)	18.0 ± 0.03	[82]
TiO_2_/BPE D	NR	NR	*Penicillum chrysogenum* (MTCC 5108)	15.0 ± 0.04	[82]
TiO_2_/BPE E	NR	NR	*Penicillum chrysogenum* (MTCC 5108)	13.5 ± 0.08	[82]

BPE, Brahmi plant extract; NR, Not reported.

**Table 6:** Activities of NPs tested on different fungal species expressed as a percentage.

Type of NP	Shape	Size (nm)	Organism(s) Tested	Activity (%)	Reference
Ag	NR	20–100	*Cladosporium cladosporioides* *Aspergillus niger*	50 µg/mL → 90 50 µg/mL → 70	[83]
Ag	Polygonal	35 ± 15	*Candida tropicalis* *Saccharomyces boulardii*	25 µg/mL → >95 50 µg/mL → >95 25 µg/mL → <50 50 µg/mL → >95	[84]
Ag	Spherical	5	*Colletotrichum gloeosporioides*	13 µg/mL → 73 26 µg/mL → 82 56 µg/mL → 89	[85]
Ag	Spherical	24	*Colletotrichum gloeosporioides*	13 µg/mL → 74 26 µg/mL → 82 56 µg/mL → 89	[85]
ZnO	Spherical	30–45	*Erythricium salmonicolor*	12 mmol/L, day 7 → 71.3 12 mmol/L, day 10 → 51.1 9 mmol/L, day 7 → 58.3 9 mmol/L, day 10 → 44.6 6 mmol/L, day 7 → 48.9 6 mmol/L, day 10 → 23.6 3 mmol/L, day 7 → 36.9 3 mmol/L, day 10 → 14.1	[86]
Ag	NR	NR	*Rhizoctonia solani* (AG1) *Rhizoctonia solani* (AG4) *Macrophomina phaseolina* *Sclerotinia sclerotiorum* *Trichoderma harzianum* *Pythium aphanidermatum*	6 µg/mL → 75 8 µg/mL → 80 10 µg/mL → 90 12 µg/mL → 90 14 µg/mL → 90 16 µg/mL → 100 6 µg/mL → ≥90 8 µg/mL → ≥90 10 µg/mL → ≥90 12 µg/mL → 100 14 µg/mL → 100 16 µg/mL → 100 6 µg/mL → 100 8 µg/mL → 100 10 µg/mL → 100 12 µg/mL → 100 14 µg/mL → 100 16 µg/mL → 100 6 µg/mL → ≥95 8 µg/mL → 100 10 µg/mL → 100 12 µg/mL → 100 14 µg/mL → 100 16 µg/mL → 100 6 µg/mL → 80 8 µg/mL → 84 10 µg/mL → 90 12 µg/mL → 100 14 µg/mL → 100 16 µg/mL → 100 6 µg/mL → 100 8 µg/mL → 100 10 µg/mL → 100 12 µg/mL → 100 14 µg/mL → 100 16 µg/mL → 100	[87]
Ag	Spherical	10–20	*Bipolaris sorokiniana*	≥2 µg/mL → 100	[88]
Ag	Spherical	1–9	*Aspergillus flavus*	5 µg/mL → 0 15 µg/mL → 30 25 µg/mL → 58 35 µg/mL → 85 45 µg/mL → 98 60 µg/mL → 100	[89]
PEI^1^/Ag	Spherical	20.6	*Rhizopus arrhizus*	1.6 µg/mL → 97	[90]
PEI^2^/Ag	Spherical	4.24		6.5 µg/mL → 94	
SiO_2_	Spherical	9.92–19.8	*Rhizoctonia solani*	100 µg/mL → 93–100	[91]

NR, Not reported; PEI^1^, polyethyleneimine 700,000 Da; PEI^2^, polyethyleneimine 65,000 Da.

**Table 7:** Current antifungal classes and their mechanisms of action.

Class	Examples	Mechanism of Action
Allylamines	Terbinafine Naftifine	Squalene epoxidase inhibition, responsible for conversion of squalene to ergosterol
Azoles	Clotrimazole Miconazole Ketoconazole Fluconazole Itrazonacole	C14-α demethylation inhibition of lanosterol, inhibiting ergosterol synthesis
Echinocandins	Caspofungin Micafungin Anidulafungin	β-(1,3)-D-glucan synthase inhibition, interfering with cell wall synthesis
Polyenes	Amphotericin B Nystatin Candicidin	Ergosterol binding, forming pores and causing leakage, inhibiting proper transport mechanisms
Antimetabolites	Flucytosine	Pyrimidine analogue, interfering with nucleic acid synthesis
Triterpenoids	Ibrexafungerp	β-(1,3)-D-glucan synthase inhibition, interfering with cell wall synthesis

## Data Availability

All data is available upon request.
